# Fibular osteotomy is helpful for talar reduction in the treatment of varus ankle osteoarthritis with supramalleolar osteotomy

**DOI:** 10.1186/s13018-021-02732-8

**Published:** 2021-09-26

**Authors:** Jing-Qi Liang, Jun-Hu Wang, Yan Zhang, Xiao-Dong Wen, Pei-Long Liu, Xiao-Jun Liang, Jun Lu, Yi Li, Hong-Mou Zhao

**Affiliations:** Foot and Ankle Surgery Department, Honghui Hospital of Xi’an Jiaotong University, No. 76 Nanguo Road, Xi’an, 710054 People’s Republic of China

**Keywords:** Ankle osteoarthritis, Realignment surgery, Supramalleolar osteotomy, Fibular osteotomy

## Abstract

**Background:**

There have been debates on the necessity of fibular osteotomy (FO) in supramalleolar osteotomy (SMOT) for the treatment of varus ankle osteoarthritis. The purpose of the current study was to compare the clinical and radiological outcomes between SMOT with and without FO in the treatment of varus ankle osteoarthritis.

**Methods:**

The SMOT group included 39 patients, and the SMOT with FO group included 24 patients. The basic information reached no significant difference between groups. The American Orthopedic Foot and Ankle Society (AOFAS) ankle-hindfoot score, Ankle Osteoarthritis Scale (AOS), modified Takakura stage and range of motion (ROM) were used for the functional evaluation. The radiologic parameters were assessed at the last follow-up to compare the degree of talar reduction between the two groups.

**Results:**

Both groups achieved significant improvements in AOFAS scores, modified Takakura stage, as well as AOS pain and functional scores (*P* < 0.001). The ROM of the ankle joint in the SMOT group was significantly decreased (*P* = 0.022). In both groups, all of the radiological parameters were significantly improved (*P* < 0.01). The tibiofibular clear space (TFCS) was significantly widened in the SMOT group (*P* < 0.001). No significant difference was found between the two groups according to the functional outcomes. However, the talar tilt angle (TT) and hindfoot alignment angle (HFA) in the SMOT with FO group were significantly smaller than those in the SMOT group (*P* < 0.05). The TFCS was significantly widened in the SMOT group (*P* = 0.001). The medial displacement of the talus (MDT) was better reduced in the SMOT with FO group (*P* = 0.006).

**Conclusion:**

SMOT is a promising procedure for functional improvement and malalignment correction in varus ankle osteoarthritis but reduces ankle range of motion. If SMOT is combined with FO, talar tilt and medial displacement will be better reduced.

## Background

Unlike the hip and knee, which are prone to developing primary osteoarthritis, the ankle develops arthritis usually because of a traumatic event, mainly ankle sprains. Lateral loosening and instability lead to medial stress concentration, and uneven pressure on the articular surface is closely related to cartilage degeneration, which may induce osteoarthritis changes and progress the development of degeneration [1–3]. There is consensus on the use joint-sacrificing procedures, including total ankle replacement or ankle arthrodesis, for the treatment of painful end-stage ankle osteoarthritis [1, 4]. However, more than half of the tibiotalar joint surface is usually preserved in early and mid-stage ankle osteoarthritis, and treatment is challenging and controversial [5].

Supramalleolar osteotomy (SMOT) is an effective procedure for the treatment of non-end-stage asymmetric ankle osteoarthritis that was first introduced by Speed and Boyd in 1936 [6] and was popularized after Takakura’s report in 1995 [7]. Clinical and biomechanical studies have reported that SMOT can realign the weight-bearing axis, restore the congruence of the tibiotalar joint [8–12], decrease the contact pressure of the medial malleolar joint [13, 14], and even reverse the stage of radiological ankle osteoarthritis [5, 7, 15]. However, the indications for this procedure are still controversial, and some patients have reached unsatisfactory outcomes.

The necessity of fibula osteotomy (FO) is one of the most controversial issues. Some authors have proposed the combination of SMOT and FO in all cases [7, 10, 15–18], some have suggested that the entirety of the fibula should be reserved [8, 19–21], and some have used FO depending on the conditions [5, 9, 12, 22, 23]. However, until now, the indications for FO have remained unclear. Thus, we hypothesize that FO may play a role in realignment surgery and may be helpful in restoring the congruence of the ankle joint. The purpose of the current study is to retrospectively analyze and compare the clinical and radiological outcomes of SMOT with or without FO for the treatment of varus ankle osteoarthritis.

## Methods

The current study was approved by the research board of our hospital. The authors retrospectively studied the outcomes of SMOT with or without FO in the treatment of varus ankle osteoarthritis between January 2010 and January 2018. The inclusion criteria were as follows: (1) adults more than 18 years old; (2) tibial articular surface angle (TAS) less than 84 degrees; (3) varus ankle osteoarthritis; (4) clinical symptoms, such as pain with walking and limitations in daily and recreational activities; (5) treated with SMOT with or without FO; and (6) at least two years of follow-up. The exclusion criteria were as follows: patients with (1) neurological disorders; (2) rheumatoid arthritis; (3) Charcot arthropathy; (4) Charcot-Marry-Tooth deformity; (5) acute or chronic infections of the ankle joint; or (6) resurgery after SMOT failure.

Finally, 39 patients in the SMOT group and 24 patients in the SMOT with FO group were enrolled in the study. There were 19 males and 44 females, and the mean age was 55.7 ± 9.4 (range, 23–77) years. According to the modified Takakura ankle osteoarthritis stage, there were 9 stage 2, 25 stage 3a, 27 stage 3b and 2 stage 4 patients. The basic information of the included patients is listed in Table 1, and no significant differences were found with the numbers available.

**Table 1:** Basic information and preoperative parameters of the two groups

	SMOT (*n* = 39)	SMOT with FO (*n* = 24)	*P* value
Male/female	13/26	6/18	0.484
Age, year	55.3 ± 9.8	56.4 ± 9.1	0.658
Left/right	15/24	10/14	0.801
Brostrom procedure	17	16	0.075
Calcaneal osteotomy	2	2	0.632
Takakura stage 2/3a/3b/4	7/16/15/1	2/9/12/1	0.662
Auto-/allograft	8/31	7/17	0.434
Follow-up, month	44.4 ± 20.5	49.2 ± 18.9	0.357
*Preoperative outcomes*			
AOFAS score, point	50.5 ± 11.2	48.2 ± 14.4	0.481
AOS pain, point	5.7 ± 0.9	5.6 ± 1.0	0.683
AOS function, point	6.1 ± 0.9	5.7 ± 1.1	0.121
ROM of ankle, degree	40.1 ± 8.4	37.8 ± 9.2	0.313
*Preoperative radiological parameters*			
TAS, degree	81.8 ± 4.1	80.1 ± 3.5	0.097
TT, degree	6.9 ± 4.7	7.1 ± 4.9	0.872
TMM, degree	34.4 ± 6.7	33.7 ± 5.7	0.672
TC, degree	70.2 ± 3.9	68.7 ± 4.3	0.159
TLS, degree	75.2 ± 3.2	74.3 ± 3.8	0.317
HFA^a^, degree	14.8 ± 3.4	15.9 ± 5.1	0.308
TFCS, mm	2.9 ± 0.5	2.8 ± 0.6	0.478
MDT, mm	6.2 ± 2.0	7.2 ± 2.2	0.068

SMOT, Supramalleolar osteotomy; FO, fibular osteotomy; AOFAS, the American Orthopaedic Foot and Ankle Society ankle-hindfoot score; AOS, Ankle Osteoarthritis Scale; ROM, range of motion; TAS, tibial articular surface angle; TT, talar tilt angle; TMM, tibial medial malleolar angle; TC, tibiocrural angle; TLS, tibial lateral surface angle; HFA, hindfoot alignment angle; TFCS, tibiofibular clear space; MDT, medial displacement of talus

^a^The case number in SMOT group was 21, and in SMOT with FO group was 17

### Operative technique

All of the included patients were treated with medial opening wedge SMOT. FO was used for varus ankle osteoarthritis according to the doctors’ preference. Tibial osteotomy was performed approximately 5 cm proximal to the tip of the medial malleolus. Before osteotomy, a K-wire was placed from the medial to the lateral cortex to guide the osteotomy. The osteotomy plane was slightly inclined from medial-superior to lateral-inferior and ended at the level of syndesmosis. Subsequently, the osteotomy was performed using a wide saw blade, and the lateral cortex was carefully preserved. According to the preoperative plan, the aim for the TAS was 90 to 92 degrees, and that for the tibial lateral surface angle (TLS) was 80 to 85 degrees. An iliac autograft or allograft was used to fill the tibial osteotomy site according to the patient’s choice. The osteotomy site was internally fixed with the use of a medial plate.

**Fig. 1:**
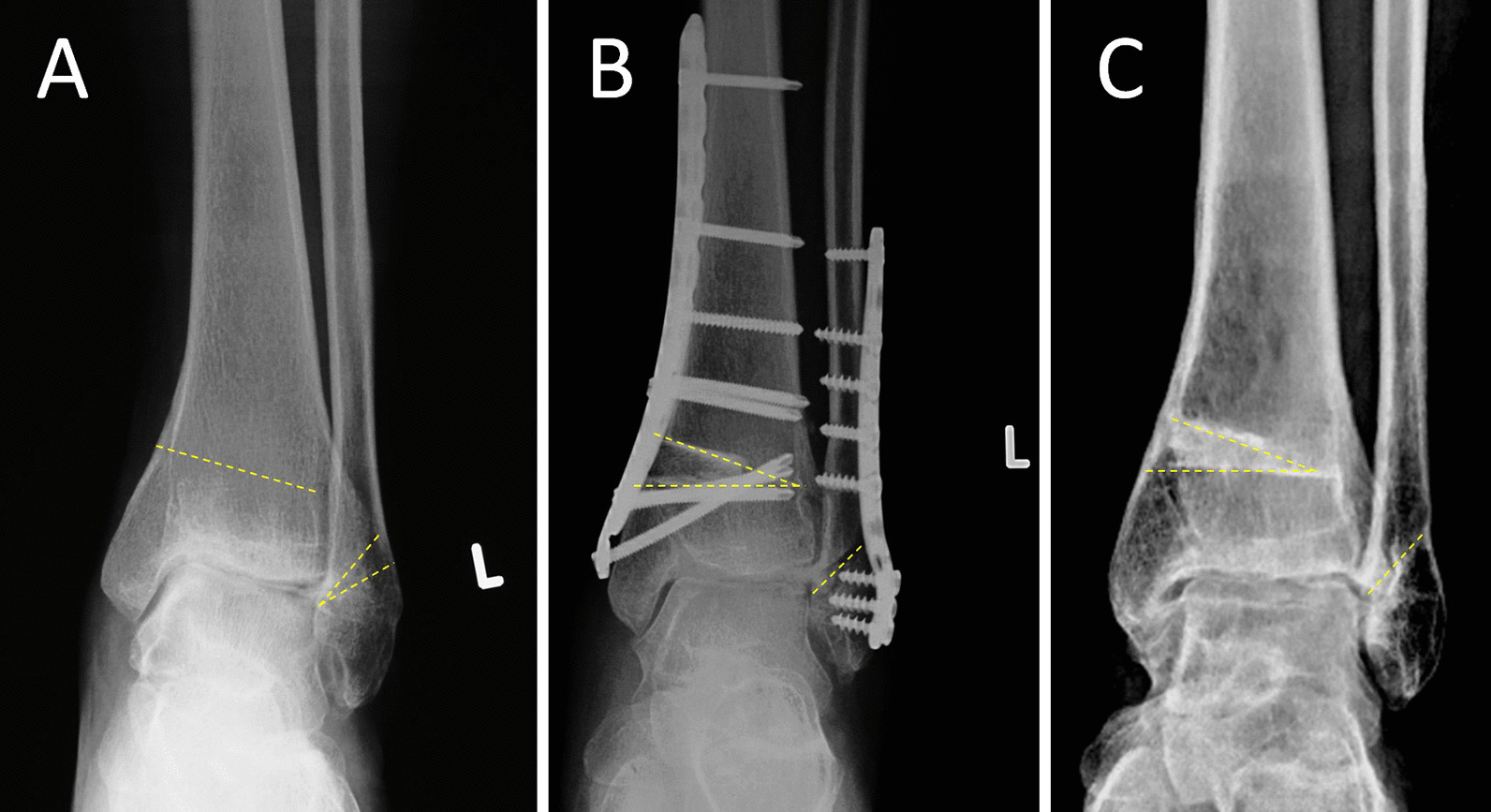
A 30-year-old female with supination-adduction ankle fracture malunion and traumatic varus ankle osteoarthritis (**a**). This patient was treated with SMOT and FO (**b**), and the 78-month follow-up results showed normal alignment (**c**)

**Fig. 2:**
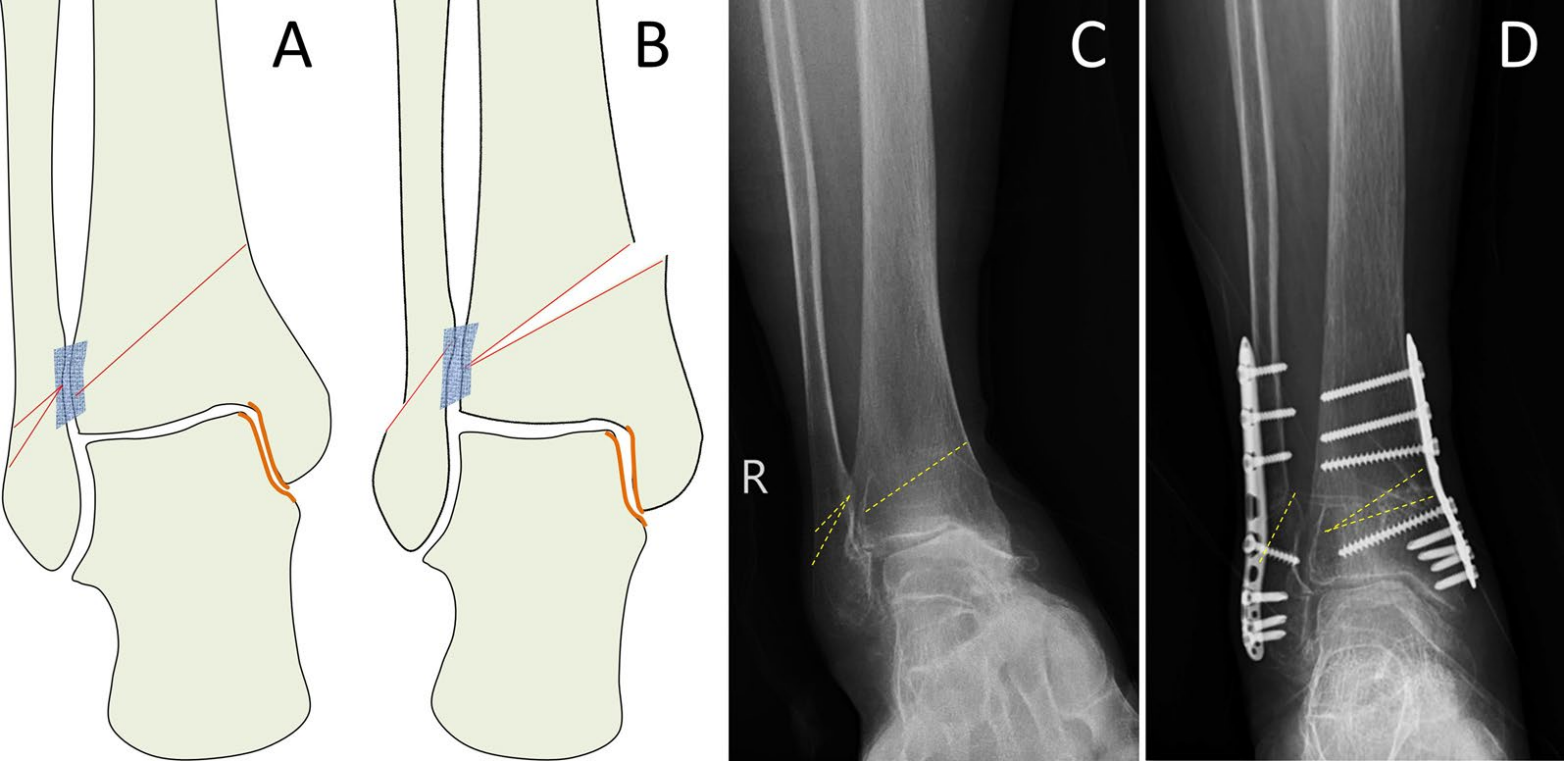
Schematic diagram of lateral close wedge osteotomy in the distal fibula (**a**, **b**). The preoperative view shows stage 3a varus ankle osteoarthritis (**c**) and treatment with SMOT and lateral close wedge FO. The 26-month postoperative view showed normal alignment, the talus moved laterally, the medial ankle joint space was widened, and talar tilt was corrected (**d**)

**Fig. 3:**
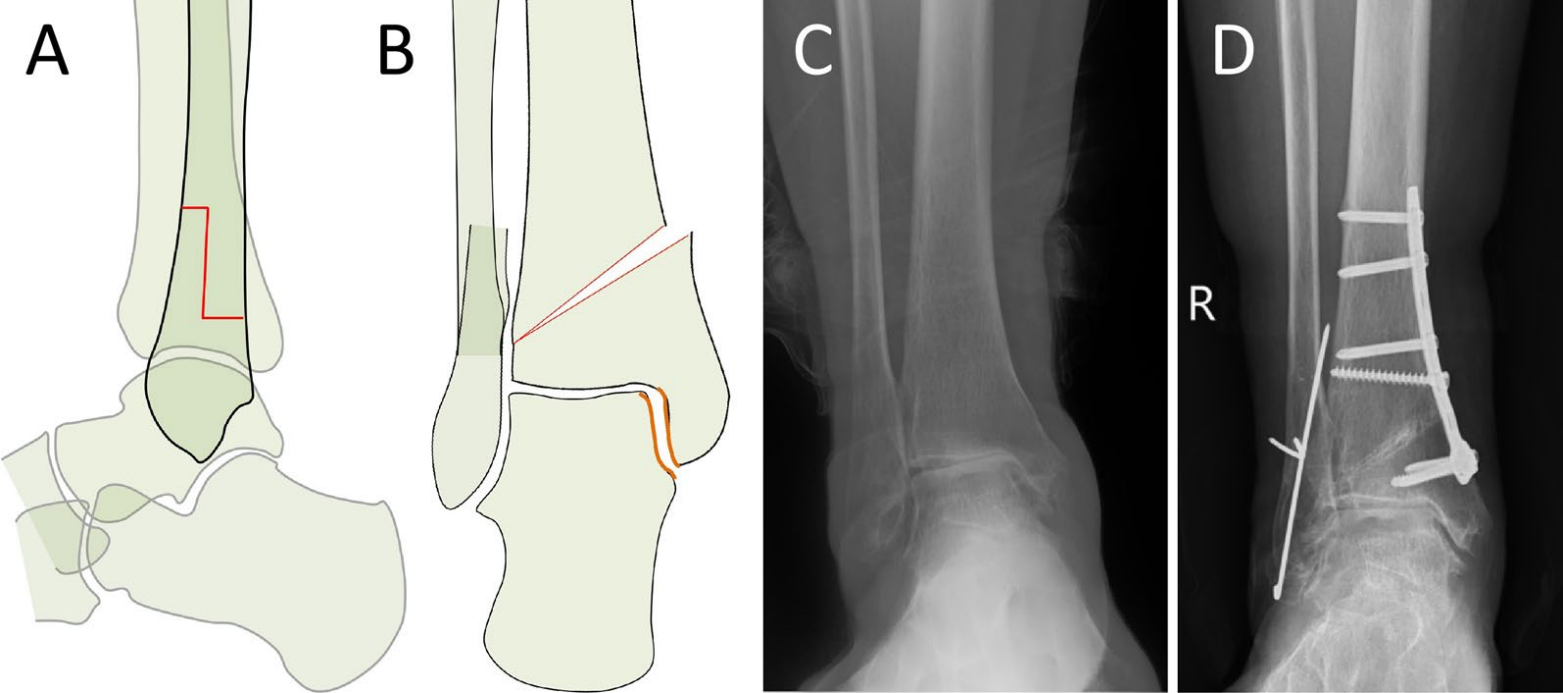
Schematic diagram of Z-shaped osteotomy in the distal fibula (**a**, **b**). The preoperative view shows stage 3a varus ankle osteoarthritis (**c**) and treatment with SMOT and Z-shaped FO. The 24-month postoperative view shows normal alignment, the talus moved laterally, the medial malleolar space was widened, and talar tilt was corrected (**d**)

FO was performed with a lateral approach at the same level or slightly higher than the tibial osteotomy level. If the patient had distal fibular fracture malunion, FO was performed at the level of the old fracture line (Fig. 1). If the fibula was normal, lateral closing wedge osteotomy (Fig. 2) or Z-shaped osteotomy was used, similar to Scarf osteotomy for hallux valgus (Fig. 3). The fibular osteotomy sites were internally fixed with plates or K-wires.

If the patient had chronic ankle joint instability or was unstable after lateral osteophyte debridement, a modified Brostrom procedure was used to enforce the lateral stability of the ankle joint. If the patient still had varus deformity of the hindfoot after SMOT, calcaneal osteotomy was used to further move the hindfoot weight-bearing site laterally.

The postoperative rehabilitation protocol included active and passive motion exercises of the ankle and forefoot joints, isotonic and isometric exercises of the leg, and a night splint, beginning on the second postoperative day in both groups. Patients were permitted to practice partial weight bearing 6 to 8 weeks postoperation. Full weight-bearing began after the osteotomy site reached bony union radiographically.

### Evaluations

The X-ray follow-up protocol included the following: during the first 3 months postoperation, anterior–posterior and lateral views of the ankle joint were used for the evaluation of bony union; weight-bearing anterior–posterior and lateral views of the ankle joint and Saltzman views were used every six months during the later follow-up time.

**Fig. 4:**
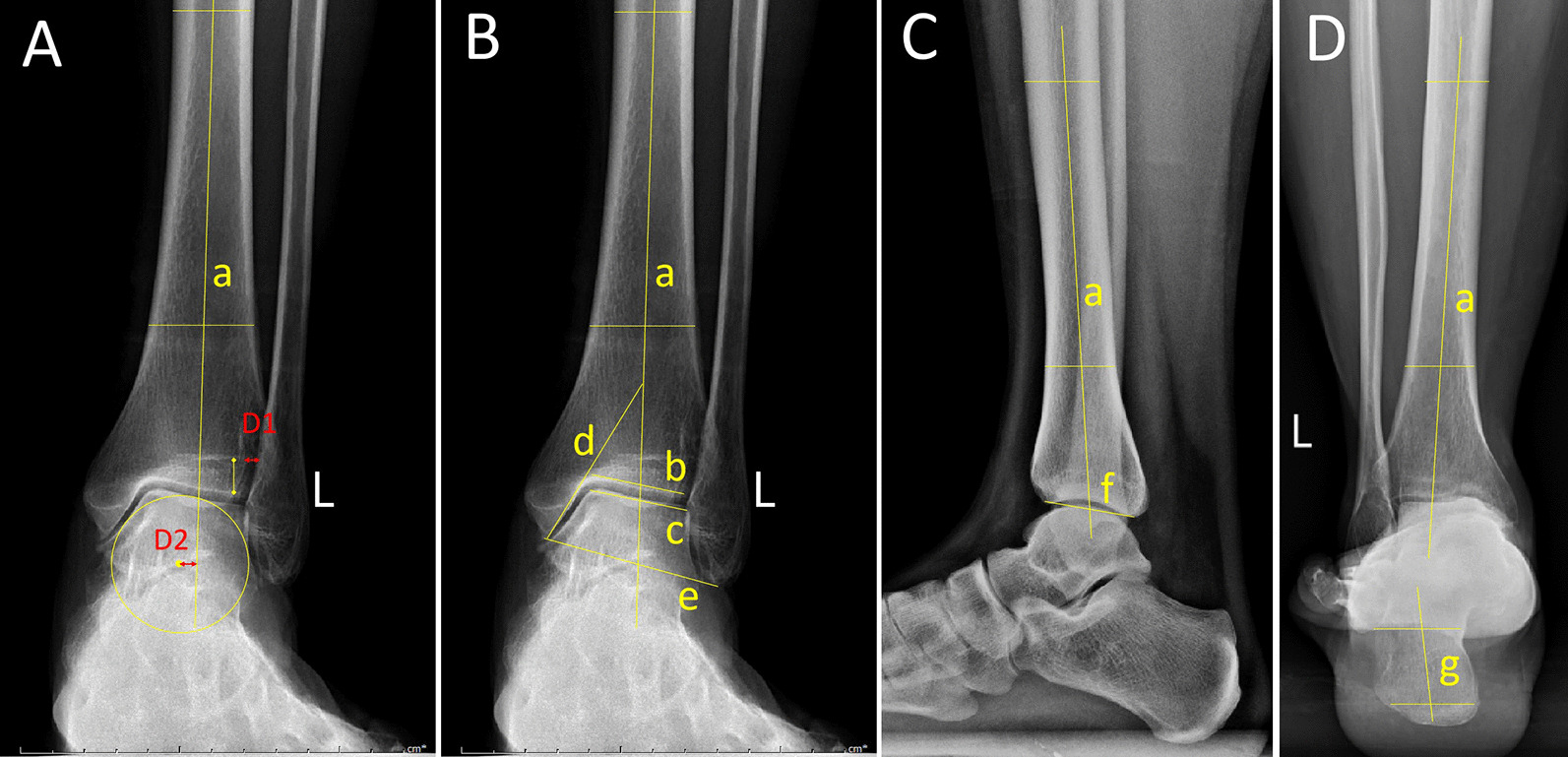
Radiological parameters used in the current study. D1 is the tibiofibular clear space (TFCS); D2 is the medial displacement of the talus (MDT) (**a**). Tibial articular surface angle (TAS), the angle between lines a and b; talar tilt angle (TT), the angle between lines b and c; tibiocrural angle (TC), the angle between lines a and e; tibial medial malleolar angle (TMM), the angle between lines a and d (**b**). Tibial lateral surface angle (TLS), the angle between lines a and f (**c**). The hindfoot alignment angle (HFA), the line between a and g (**d**)

The tibiofibular clear space (TFCS) is the distance of inferior tibiofibular syndesmosis, measured at a level 1 cm proximal to the distal tibial articular surface, normal less than 5 mm [24] (Fig. 4a). The medial displacement of the talus (MDT) is the distance from the center of the talus to the axis of the tibia (Fig. 4a). Other radiological parameters included the tibial articular surface angle (TAS), talar tilt angle (TT), tibiocrural angle (TC), and tibial medial malleolar angle (TMM) on anterior–posterior views (Fig. 4b); the tibial lateral surface (TLS) angle on lateral views (Fig. 4c); and the hindfoot alignment angle (HFA) on Saltzman views (Fig. 4d). All of the included measurements on weight-bearing radiographs were performed by two observers independently, and the mean of the two observers was used as the result.

The American Orthopedic Foot and Ankle Society (AOFAS) ankle-hindfoot score and the Ankle Osteoarthritis Scale (AOS) were used to evaluate the functional outcomes pre- and postoperation [25, 26]. Treatment failure was defined as a patient who required resurgery for reasons related to the operation. Resurgeries included osteotomy, arthrodesis, and arthroplasty, and patients with no symptoms due to hardware removal were not included. The pre- and postoperative range of motion (ROM) values of the ankle joint were recorded. The functional outcomes and radiological parameters before failure relative to resurgery were included as the patients’ final results.

### Statistical analyses

Descriptive statistics were calculated as the mean ± SD. Statistical analyses of the included data were performed using Student’s *t* test, Pearson’s chi-square test or Fisher’s exact test with the level of significance set at *α* = 0.05. The statistical analyses were performed with SPSS 17.0 software (SPSS Inc, Chicago, Illinois).

## Results

### Functional and radiological improvements in the two groups

All patients in both groups reached bony union, without incision-related complications. Both groups achieved significant improvements in AOFAS scores, AOS pain and functional scores, and modified Takakura stage (*P* < 0.001, Table 2), compared with the preoperative values. The ROM in the SMOT group was significantly decreased (*P* = 0.022) but reached no significant difference in the SMOT with FO group with the numbers available.

**Table 2:** Comparison of the preoperative and last follow-up time functional outcomes and radiological parameters

	SMOT (*n* = 39)		*P* value	SMOT with FO (*n* = 24)		*P* value
	Preoperation	Last follow-up		Preoperation	Last follow-up	
*Functional outcomes*						
AOFAS, point	50.5 ± 11.2	79.1 ± 12.4	0.000	48.2 ± 14.4	83.2 ± 10.3	0.000
AOS pain, point	5.7 ± 0.9	2.9 ± 1.1	0.000	5.6 ± 1.0	2.6 ± 0.9	0.000
AOS function, point	6.1 ± 0.9	3.0 ± 1.3	0.000	5.7 ± 1.1	2.9 ± 1.2	0.000
ROM of ankle, degree	40.1 ± 8.4	36.5 ± 4.7	0.022	37.8 ± 9.2	39.3 ± 7.4	0.537
Takakura stage 1/2/3a/3b/4	0/7/16/15/1	26/9/2/2/0	0.000	0/2/9/12/1	19/3/1/1/0	0.000
*Radiological parameters*						
TAS, degree	81.8 ± 4.1	89.0 ± 3.3	0.000	80.1 ± 3.5	89.9 ± 2.8	0.000
TT, degree	6.9 ± 4.7	2.6 ± 1.4	0.000	7.1 ± 4.9	1.9 ± 0.9	0.000
TMM, degree	34.4 ± 6.7	26.5 ± 3.6	0.000	33.7 ± 5.7	28.1 ± 5.5	0.001
TC, degree	70.2 ± 3.9	76.9 ± 4.5	0.000	68.7 ± 4.3	78.6 ± 4.1	0.000
TLS, degree	75.2 ± 3.2	79.7 ± 2.9	0.000	74.3 ± 3.8	80.9 ± 1.8	0.000
HFA^a^, degree	14.8 ± 3.4	4.1 ± 2.0	0.000	15.9 ± 5.1	2.9 ± 1.4	0.000
TFCS, mm	2.9 ± 0.5	4.2 ± 1.1	0.000	2.8 ± 0.6	3.2 ± 0.8	0.056
MDT, mm	6.2 ± 2.0	3.1 ± 1.6	0.000	7.2 ± 2.2	1.9 ± 1.7	0.000

SMOT, Supramalleolar osteotomy; FO, fibular osteotomy; AOFAS, the American Orthopaedic Foot and Ankle Society ankle-hindfoot score; AOS, Ankle Osteoarthritis Scale; ROM, range of motion; TAS, tibial articular surface angle; TT, talar tilt angle; TMM, tibial medial malleolar angle; TC, tibiocrural angle; TLS, tibial lateral surface angle; HFA, hindfoot alignment angle; TFCS, tibiofibular clear space; MDT, medial displacement of talus

^a^The case number in SMOT group was 21, and in SMOT with FO group was 17

In both groups, the radiological parameters TAS, TT, TMM, TC, TLS, HFA, and MDT were all significantly improved (*P* < 0.01). The TFCS was significantly widened in the SMOT group (*P* < 0.001) but reached no significant difference in the SMOT with FO group with the numbers available.

### Functional and radiological comparison between the two groups

While comparing the postoperative functional outcomes of the two groups, the AOFAS scores, AOS pain and function scores, modified Takakura stage and ROM values did not reach statistical significance at the final follow-up time with the numbers available (Table 3). In the SMOT group, seven patients experienced failure: three patients underwent ankle arthrodesis at 17, 26, and 61 months because of pain and dysfunction, one patient was treated with fibular osteotomy, and three patients made appointments for fibular osteotomy. One patient in the SMOT with FO group needed arthrodesis at 14 months. However, the failure rate was not significantly different with the numbers available.

**Table 3:** Functional outcomes and radiological parameters between the two groups at the last follow-up time

	SMOT (*n* = 39)	SMOT with FO (*n* = 24)	*P* value
*Functional outcomes*			
AOFAS, point	79.1 ± 12.4	83.2 ± 10.3	0.180
AOS pain, point	2.9 ± 1.1	2.6 ± 0.9	0.266
AOS function, point	3.0 ± 1.3	2.9 ± 1.2	0.761
ROM of ankle, degree	36.5 ± 4.7	39.3 ± 7.4	0.071
Takakura stage 1/2/3a/3b/4	26/9/2/2/0	19/3/1/1/0	0.740
Failure rate	17.9% (7/39)	4.2% (1/24)	0.141
*Radiological parameters*			
TAS	89.0 ± 3.3	89.9 ± 2.8	0.271
TT	2.6 ± 1.4	1.9 ± 0.9	0.033
TMM	26.5 ± 3.6	28.1 ± 5.5	0.167
TC	76.9 ± 4.5	78.6 ± 4.1	0.137
TLS	79.7 ± 2.9	80.9 ± 1.8	0.074
HFA^a^	4.1 ± 2.0	2.9 ± 1.4	0.043
TFCS	4.2 ± 1.1	3.3 ± 0.8	0.001
MDT	3.1 ± 1.6	1.9 ± 1.7	0.006

SMOT, Supramalleolar osteotomy; FO, fibular osteotomy; AOFAS, the American Orthopaedic Foot and Ankle Society ankle-hindfoot score; AOS, Ankle Osteoarthritis Scale; ROM, range of motion; TAS, tibial articular surface angle; TT, talar tilt angle; TMM, tibial medial malleolar angle; TC, tibiocrural angle; TLS, tibial lateral surface angle; HFA, hindfoot alignment angle; TFCS, tibiofibular clear space; MDT, medial displacement of talus

^a^The case number in SMOT group was 21, and in SMOT with FO group was 17

When comparing the postoperative radiological parameters of the two groups, the TT angle in the SMOT with FO group was significantly smaller than that in the SMOT group (*P* = 0.033). The HFA in the SMOT with FO group was significantly smaller than that in the SMOT group (*P* = 0.043). The TFCS was significantly widened in the SMOT group (*P* = 0.001). The MDT was better reduced in the SMOT with FO group (*P* = 0.006). The other postoperative radiological parameters, which included the TAS, TMM, TC, and TLS, were not significantly different with the numbers available.

## Discussion

Realignment surgery, which is based on the theory that uneven pressure on the articular surface of the lower extremities may induce degeneration [3], is used to redistribute the joint weight-bearing pressure to delay the progression of osteoarthritis. The midterm results of SMOT showed good outcomes for pain relief, functional improvements, and returning to sports and recreation activities [7–12, 15–19, 21, 23, 27, 28]. However, SMOT is still controversial, the indications for this procedure are still unclear, and the evidence is limited.

**Fig. 5:**
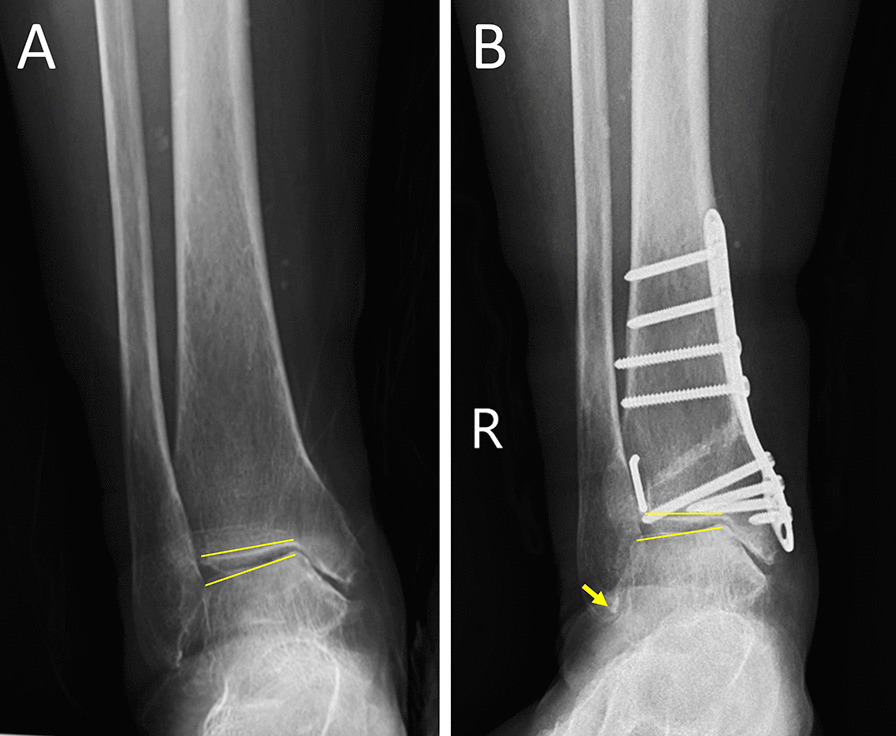
A 58-year-old male patient. The preoperative anterior–posterior view (**a**) shows stage 2 varus ankle osteoarthritis. The postoperative anterior–posterior view shows the talar tilt was not corrected and impingement of the distal fibula (yellow arrow) (**b**)

When the SMOT procedure was used for varus ankle osteoarthritis, the necessity of FO was debated. Takakura and Tanaka performed FO first before the SMOT procedure in all cases [7, 15]. Stamatis et al. [18] also performed FO first and at the same level as the tibial osteotomy level without any internal fixation. These earlier studies used tibial osteotomy at the level proximal to the level of syndesmosis; while tibial osteotomy was gradually carried out, the distal part of the tibia was rotated with the lateral cortex of the tibia as the center of rotation. In this case, syndesmosis may impact and prevent the rotation, and FO may be a necessary procedure to facilitate the medial opening of the tibia. Ahn et al. [8] reported distal tibial oblique osteotomy without FO to constrict the ankle mortise and to achieve a lateral shift of the weight-bearing center of the talus. His tibial osteotomy line was on the lateral tibial cortex 5 mm proximal to the joint and distal to the syndesmosis to avoid syndesmosis impingement. However, in his cases, talar tilt was not corrected. In our patients treated with SMOT and FO, talar tilt was better corrected than in those without FO (Fig. 5). This result suggests that the fibula plays a role in hindering the reduction in the talus. Biomechanical studies have reported that creating a supramalleolar valgus deformity does not cause a shift in contact toward the lateral side of the tibiotalar joint, and the restrictive role of the fibula is revealed during osteotomy [13, 14]. According to current results, the weight-bearing center of the talus was better reduced in the SMOT with FO group (*P* = 0.006). In current study, we used lateral closing wedge or Z-shaped FO at the same level to the tibial osteotomy. Although the osteotomy methods were different, they played the same roles to relieve the lateral stress and allowed the distal tibial to rotate centered on the lateral cortex when the osteotomy site was opened.

**Fig. 6:**
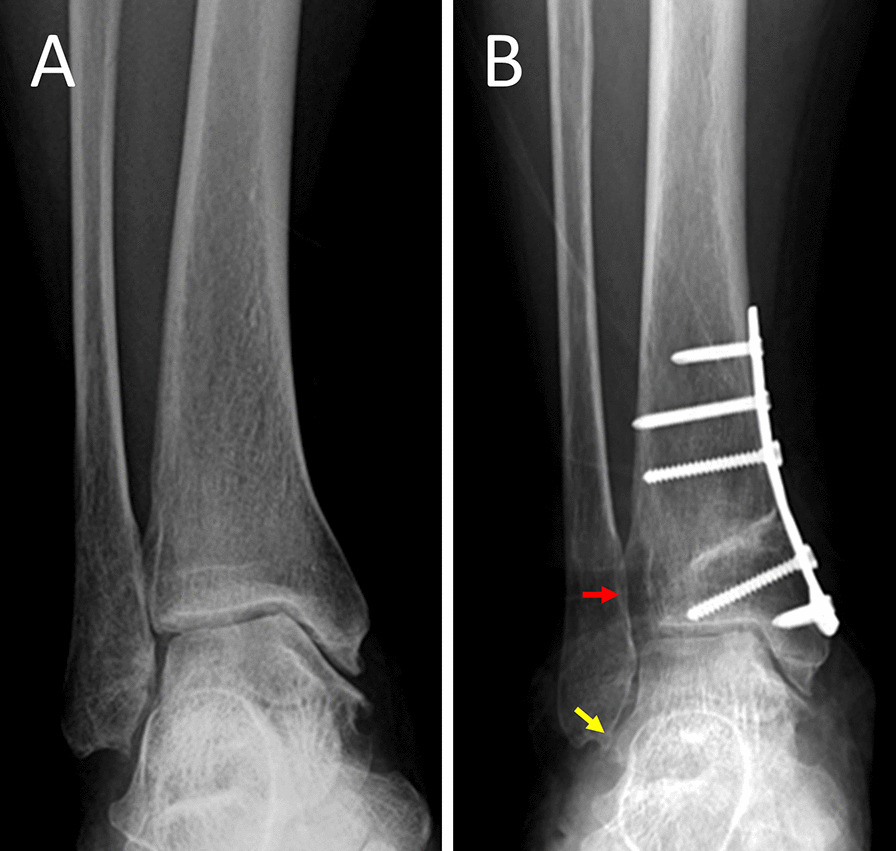
A 58-year-old male patient. The preoperative anterior–posterior view (**a**) shows stage 2 varus ankle osteoarthritis. The postoperative anterior–posterior view shows widening of the distal tibiofibular syndesmosis (red arrow) and impingement of the distal fibula (yellow arrow) (**b**)

In SMOT without FO, the ankle mortise may be narrowed. As the talus dome has an anatomy of a wide anterior and narrow posterior, dorsiflexion of the ankle joint may be limited after the SMOT procedure. Pagenstert et al. [23] reported that the improvement of pain was correlated with walking ability and general activity but was not correlated with the achieved ROM. Nüesch et al. [11] reported that after SMOT, patients walked more slowly, had a smaller sagittal range of motion in their affected leg, and had lower peak ankle dorsiflexion; however, they observed no difference in the quality-of-life score compared with the healthy controls. According to the current results, the ROM in SMOT decreased postoperation, although the decreased ROM was not reported to influence the physical activity of the SMOT patients. Long-term weight bearing and dorsiflexion may concentrate the stress on syndesmosis and widen the distal tibiofibular syndesmosis (Fig. 6). This narrowing of the mortise may lead to lateral malleolar impingement during weight bearing and walking and may induce pain symptoms. In our cases without FO, seven patients experienced failure: three patients with ankle arthrodesis and four patients who made an appointment for fibular osteotomy.

There are limitations in this study. First, the retrospective design led to some differences between the two groups, and there were more patients in the SMOT group. However, other basic information was not significantly different between the two groups, which makes the results of the two groups comparable. Second, there were some additional procedures, such as modified Brostrom procedure or calcaneal osteotomy, that might affect the final outcomes of SMOT. However, there was no significant difference in the proportion of additional procedures between the two groups (Table 1). The third limitation is that the follow-up period was short, with a mean time of 46 months. Although the outcomes will change over time, our early results confirm that the functional outcome of SMOT is good in terms of pain relief, correction of malalignment, and a reduction in symptoms for varus ankle osteoarthritis patients. Moreover, if SMOT is combined with the use of FO, talar tilt and medial displacement can be better corrected.

## Conclusions

In conclusion, SMOT is a promising procedure for functional improvement and malalignment correction in varus ankle osteoarthritis, but it reduces ankle range of motion. If SMOT is combined with FO, talar tilt and medial displacement can be better reduced. However, well-designed prospective comparative studies are still needed to further clarify the necessity of FO, while SMOT is used for the treatment of varus ankle osteoarthritis.

## Data Availability

The data of this study were real and were performed in the SPSS 17.0 software (SPSS Inc., Chicago, Illinois). The statistical results of the data are presented in this main paper. The images of the case examples are depicted in this research article. All of the data are available in contact with the corresponding author.

## Abbreviations

AOFAS: American orthopaedic foot and ankle society
AOS: Ankle osteoarthritis scale
FO: Fibular osteotomy
HFA: Hindfoot alignment angle
MDT: Medial displacement of talus
ROM: Range of motion
SMOT: Supramalleolar osteotomy
TAS: Tibial anterior surface angle
TC: Tibiocrural angle
TFCS: Tibiofibular clear space
TLS: Tibial lateral surface angle
TMM: Tibial medial malleolar angle
TT: Talar tilt angle
